# Neutrophil-to-platelet ratio predicts mortality following percutaneous coronary intervention in patients with acute ST-elevation myocardial infarction

**DOI:** 10.3389/fphys.2022.1011048

**Published:** 2022-09-19

**Authors:** Yuhui Lin, Wenjun Dai, Yongquan Chen, Xiaoqing He, Yunhong Xu

**Affiliations:** Department of Cardiology, The Third Affiliated Hospital of Guangzhou Medical University, Guangzhou, China

**Keywords:** neutrophil-to-platelet ratio, ST-elevation myocardial infarction, primary percutaneous coronary intervention, all-cause mortality, prognostic markers

## Abstract

This study aimed to evaluate the value of neutrophil-to-platelet ratio (NPR) in predicting all-cause mortality in patients with ST-elevation myocardial infarction (STEMI) after primary percutaneous coronary intervention (PCI). We enrolled 186 patients with STEMI who underwent primary PCI in the Third Affiliated Hospital of Guangzhou Medical University between January 2017 and December 2018. Based on the NPR values, the patients were divided into two groups: the NPR >0.035 group (n = 82) and the NPR ≤0.035 group (n = 104). All-cause mortality of the patients was followed up for 3 years. By the end of 3 years, 109 (58.6%) patients survived, 53 (28.5%) died, and 24 (12.9%) were lost to follow-up. Univariate analyses found that NPR was associated with all-cause mortality (*p* < 0.05). In COX regression analyses, patients in the high NPR group had a higher risk of all-cause death than those in the low NPR group (HR = 2.296, 95% CI: 1.150–4.582). These results indicate that NPR could predict all-cause death in 3 years after primary PCI in patients STEMI. NPR values may be useful in risk stratification and in specifying individualized treatment in patients with STEMI. In addition, NPR is a low-cost and easily accessible indicator, if its strong predictive value is confirmed in further studies of other large populations, it can be introduced into clinical practice for effective application.

## Introduction

ST-elevation myocardial infarction (STEMI) is an acute and severe form of coronary artery disease, which often leads to sudden cardiac death and post-infarction complications such as acute heart failure. Intravenous thrombolysis and percutaneous coronary intervention (PCI) have significantly improved the prognosis of patients with STEMI; however, patients underwent PCI still have significant risk of death and other cardiovascular adverse events. It is critical to identify patients at high risk of death so that special treatments can be initiated early. Unfortunately, risk stratification and prediction of post-PCI mortality in STEMI patients have always been challenging in clinical practice.

Previous studies demonstrated that inflammatory markers might be useful in risk stratification and prognostic prediction of patients with STEMI since acute inflammatory response serves as a critical pathogenetic process in acute myocardial infarction (Çiçek et al., 2015; Seferović et al., 2018). Neutrophils, as the first responders to acute inflammation, play an important role in the pathophysiology of STEMI. Recruitment of neutrophils can mediate microvascular injury, constriction, and plugging, leading to slow-flow or no-reflow phenomenon after PCI(Allencherril et al., 2019; Kaur et al., 2021). On the other hand, platelets are viewed as the mediators of chronic inflammatory reaction, and platelet counts in peripheral blood are correlated with the levels of various inflammatory markers, including C-reactive protein, interleukin 1, and interleukin 6 (Semple et al., 2011; Vieira-de-Abreu et al., 2012; Swirski, 2020) In addition, activation of platelets plays a crucial role in the development and progression of atherosclerosis (Swirski, 2020). Neutrophil-to-platelet ratio (NPR) is a novel inflammatory marker, which appropriately adjusts for the intensity of acute inflammatory response (expressed by neutrophils) while taking into account preexisting chronic inflammatory states (represented by platelets). NPR has been reported to be associated with poor prognosis of patients with acute ischemic stroke (Jin et al., 2019). However, the predictive value of NPR for outcome of patients with coronary artery disease is unknown. The present study aimed to assess the predictive value of NPR for major adverse cardiovascular events (MACE) in patients with STEMI after primary PCI.

## Materials and methods

### Subjects

We enrolled a total of 186 patients with STEMI who underwent primary PCI in the Third Affiliated Hospital of Guangzhou Medical University between January 2017 and December 2018. The study was approved by the ethics committee of the Third Affiliated Hospital of Guangzhou Medical University. All patients provided written informed consent. The average age of the patients (102 males and 84 females) was 66.37 ± 10.58 years old, ranging from 37 to 92 years old. Inclusion criteria: the patients met the diagnostic criteria for STEMI according to the guideline for Diagnosis and Treatment of STEMI in China; the patients underwent primary PCI within 12 h. Exclusion criteria: 1) complicated with fever or infectious diseases; 2) hematological disorders; 3) systemic lupus erythematosus or other autoimmune diseases; 4) had splenectomy; 5) malignant tumors; 6) severe liver dysfunction; 7) use of steroids in the past 6 months. According to Youden’s index and the results from previous studies [7], the optimal threshold for NPR to identify high-risk patients is 0.035. Therefore, the STEMI patients were divided into two groups based on their NPR values. Finally, 82 patients were divided into the high NPR group (NPR >0.035), and 104 patients were in the low NPR group (NPR ≤0.035).

### Measurements and follow-up

Peripheral venous blood was taken from all patients within 1 h after admission and before PCI for complete blood count (CBC) test. CBC tests were performed by an automatic hematology analyzer (XN9000, Sysmex Corporation, Japan). The CBC reports included counts of white blood cells, neutrophils, and platelets. The NPR value was calculated as neutrophil count/platelet count. Demographic and clinical data of the patients were collected. The patients were followed up by telephone or outpatient visiting every quarter. All-cause deaths of patients during hospitalization and within 3 years after discharge were recorded.

### Statistical analysis

Statistical analyses were performed using the SPSS 23.0 software, and *p* < 0.05 indicated statistically significant differences. Continuous variables were described as mean and standard deviation or median with interquartile range and were compared using analysis of variance test or Kruskal–Wallis test, respectively. Categorical variables were expressed as frequencies and percentages and were compared using Chi-square test. Survival rates were assessed by Kaplan-Meier survival curve and compared using log-rank test between the two groups. Multivariate Cox regression analysis was performed to evaluate the effects of NPR and other factors on all-cause mortality.

## Results

### Characteristics of patients

Demographic and clinical data of the patients in the high (n = 82) and low (n = 104) NPR groups were summarized in Table 1. Compared with patients in the low NPR group, the levels of N-terminal pro-brain natriuretic peptide (NT-proBNP), cardiac troponin I (cTnI), creatine kinase-MB (CK-MB), and low-density lipoprotein cholesterol (LDL-C), and smoking rates were significantly higher in patients in the high NPR group (*p* < 0.05 or *p* < 0.01). There were no significant differences in age, sex, percentages of diabetes or hypertension, levels of total cholesterol (TC), triglycerides (TG) and high-density lipoprotein cholesterol (HDL-C) between the two groups (all *p* > 0.05).

**TABLE 1:** Baseline characteristics of patients in the low and high NPR groups.

	High NPR (n = 82)	Low NPR (n = 104)	*p* Value
Age (years)	63 (53.7, 76)	67.5 (51, 81)	0.791
Male, n (%)	38 (46.3%)	57 (54.8%)	0.159
Smoking, n (%)	47 (57.3%)	43 (41.3%)	0.022
Diabetes, n (%)	21 (25%)	25 (24%)	0.226
Hypertension, n (%)	34 (41.5%)	41 (39.4%)	0.447
COPD, n (%)	11 (13.4%)	14 (13.5)	0.584
Atrial fibrillation, n (%)	20 (24.4%)	31 (29.8%)	0.062
Creatinine (µmol/L)	99 (74, 126.3)	122.5 (91, 147)	0.001
NT-proBNP (pg/ml)	4,197 (1,657.8, 5,941)	3,463.5 (1,450.3, 5,506.5)	<0.01
LVEF (%)	51 (42.75, 59.25)	56 (48, 62)	0.001
Heart rate (BPM)	101 (66.7, 125.3)	91.5 (74, 108)	0.034
CK-MB (U/L)	68.5 (36, 96.5)	43 (28.25, 66)	<0.01
cTNI (ng/L)	135 (66.25, 187.25)	94 (54.5, 146)	0.001
TC (mmol/L)	4.15 (2.9, 5.2)	4.55 (3.2, 5.7)	0.089
TG (mmol/L)	3.25 (2.25, 4.35)	3.05 (2.1, 4.6)	0.74
LDL-C (mmol/L)	2.99 (1.64, 3.9)	2.66 (1.23, 3.79)	0.019
HDL-C (mmol/L)	1.6 (1.0, 2.2)	1.7 (1.2, 2.2)	0.393
NPR	0.021 (0.013, 0.024)	0.095 (0.006, 0.012)	<0.01

Continuous variables are expressed as median (interquartile range)

### NPR is an independent predictor of all-cause death

**FIGURE 1:**
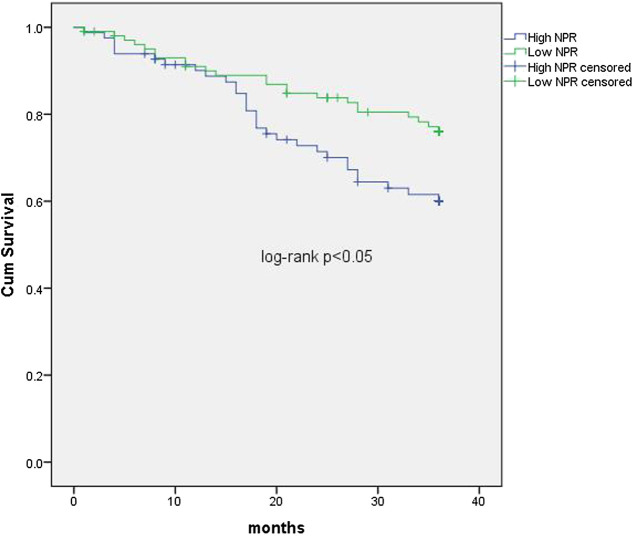
Kaplan-Meier curves comparing cumulative survival rates of patients with STEMI undergoing primary PCI in the low and high NLR ratio groups. NPR: neutrophil to platelet ratio.

**FIGURE 2:**
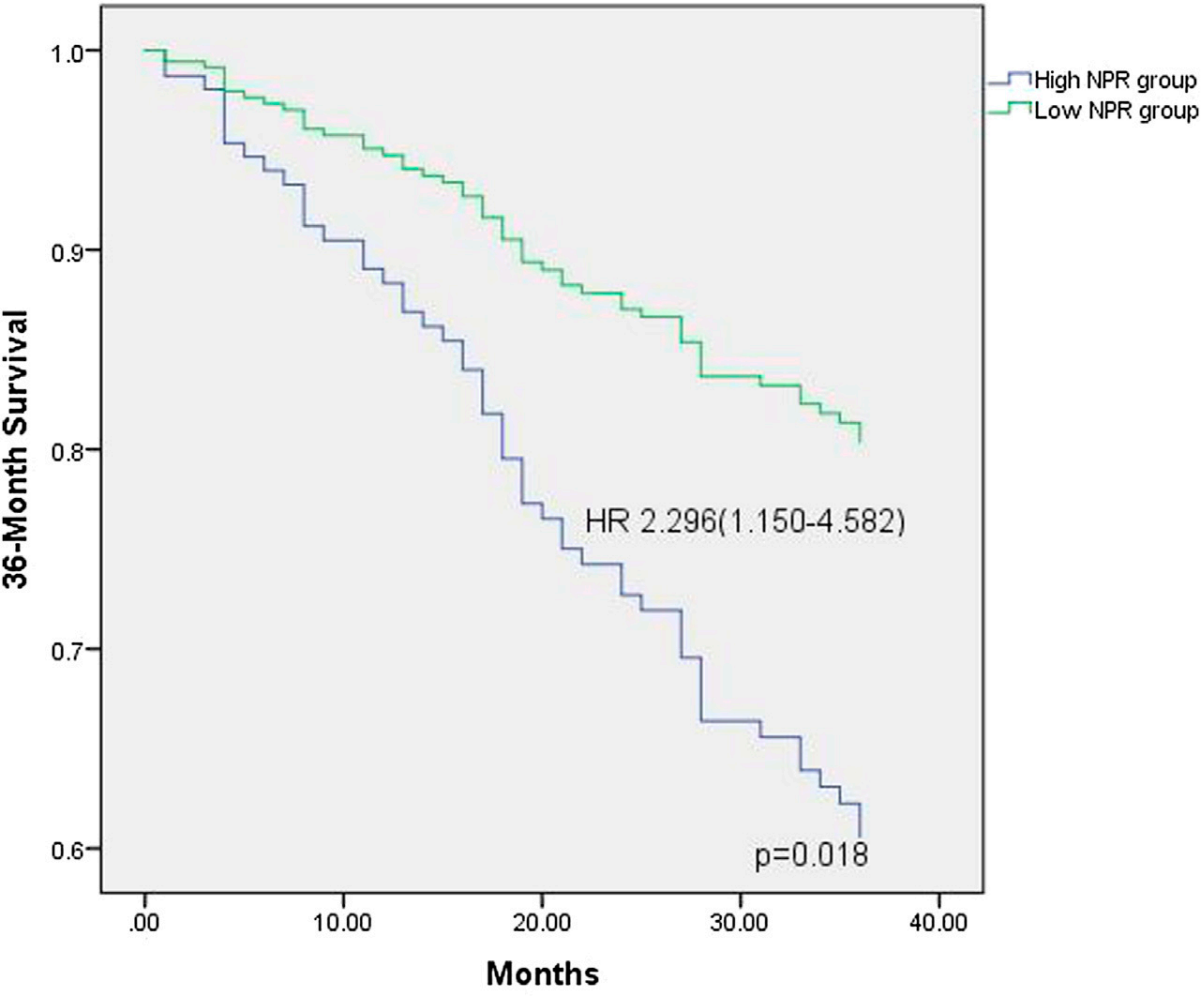
Thirty-six survival probability, stratified by NPR value, derived from multivariable Cox regression analysis (variables in the model are described in Table 2). NPR: neutrophil to platelet ratio; HR: hazard ratio.

The median follow-up period was 36 months (interquartile interval 20.75–36 months). Respectively, the median follow-up period for the low NPR group was 31.8 months and 28.9 months for the high NPR group**.** During the period, 109 (58.6%) patients survived, 53 (28.5%) died, and 24 (12.9%) were lost to follow-up. In univariate analyses, NPR was associated with all-cause death (*p* < 0.05, Figure 1). In COX regression analyses, patients in the high NPR group had a higher risk of all-cause death than those in the low NPR group (HR = 2.296, 95% CI: 1.150–4.582), *p* < 0.05, Table 2 and Figure 2).

**TABLE 2:** Cox regression model for all-cause mortality in 3 years.

Variables	*p* Value	HR (95% CI)
Age (years)	0.045	0.980 (0.961–1.000)
Male	0.969	0.989 (0.547–1.787)
Smoke	0.023	0.506 (0.281–0.912)
Diabetes mellitus	0.037	0.516 (0.278–0.959)
Hypertension	0.017	2.211 (1.150–4.2448)
COPD	0.395	0.695 (0.301–1.607)
Atrial fibrillation	0.363	1.360 (0.701–2.639)
Creatinine (µmol/L)	0.45	1.003 (0.995–1.011)
NT-proBNP	0.056	1.325 (1.103–1.639)
LVEF (%)	0.334	1.016 (0.983–1.051)
CK-MB (U/L)	0.315	0.995 (0.984–1.005)
cTNI (ng/L)	0.501	0.998 (0.994–1.003)
TC (mmol/L)	0.124	1.182 (0.995–1.461)
TG (mmol/L)	0.027	1.044 (0.837–1.294)
LDL-C (mmol/L)	0.072	1.213 (0.983–1.497)
HDL-C (mmol/L)	0.092	1.320 (0.992–1.578)
NPR >0.035	0.013	2.902 (1.253–6.722)

## Discussion

A number of clinical studies have monitored and evaluated the predicting factors of all-cause mortality of STEMI patients after discharge, and they demonstrated that delayed revascularization, multi-vessel disease, age, smoking, diabetes, hyperlipidemia, high C-reactive protein levels, and obesity are all risk factors for poor prognosis of STEMI patients (Žaliaduonytė et al., 2017). In addition to the above recognized factors, there are some novel markers including neutrophil count and platelet count may be associated with the prognosis of STEMI patients, but their value in predicting all-cause death remains elusive (Lee et al., 2012; Butt et al., 2020). Identification of novel markers that can effectively predict the risk of all-cause death after PCI in STEMI patients is of great significance for guiding early clinical intervention and improving prognosis.

The present study demonstrated that NPR was an independent predictor of all-cause death in patients with STEMI after primary PCI. Patients with higher NPR values had a significantly increased risk of all-cause death events during follow-up than patients with lower NPR values. Previous studies have shown that inflammation plays an important role in the process of atherosclerosis and thrombosis, and both neutrophils and platelets are involved in this inflammatory process (Seferović et al., 2018; Geng et al., 2019; Custodio-Chablé et al., 2020). Hypercoagulability mediated by leukocytes, especially neutrophils, and myocardial toxicity mediated by various inflammatory mediators and enzymes, such as elastase, myeloperoxidase, and acid phosphatase, contribute to adverse outcomes such as heart failure in patients with STEMI. The higher the neutrophil count, the larger the infarct size, the worse the angiographic results, and the poorer the prognosis of patients with acute STEMI(He et al., 2014). Platelets and their secreted factors are involved in many physiological and pathological reactions such as coagulation, thrombosis, inflammation, and atherosclerosis (Nording et al., 2020). Acute myocardial infarction is associated with an inflammatory response process. In patients with STEMI, the elevation in the levels of highly sensitive C-reactive protein, a marker of systemic inflammation, is associated with an increased risk of all-cause mortality events in patients with acute coronary syndrome (Munkhaugen et al., 2018). In addition, inflammation can affect platelet count and reactivity (Cornara et al., 2018). On the other hand, neutrophils are the key mediators of myocardial reperfusion injury, and neutrophil count is closely related to the infarct size and prognosis of STEMI patients undergoing primary PCI(Prompunt et al., 2018). NPR can be used as an indicator of acute inflammatory response associated with infarct size (represented by neutrophil counts) and adjusted for pre-existing chronic inflammatory states (represented by platelet counts). COX risk regression analyses were performed to further analyze the clinical data of STEMI patients, which suggest that NPR was an independent risk factor for all-cause mortality after PCI, in addition to delayed revascularization, multi-vessel disease, age, smoking, diabetes, hyperlipidemia, high C-reactive protein, and obesity.

In conclusion, NPR is a potential prognostic marker for predicting all-cause death in patients with STEMI after primary PCI. Its predictive value should be further validated in large-scale clinical trials.

## Data Availability

The raw data supporting the conclusion of this article will be made available by the authors, without undue reservation.
